# A Case of Life-Threatening Airway Obstruction Due to Epstein-Barr Virus–Associated Smooth Muscle Tumor With HIV Infection

**DOI:** 10.1093/ofid/ofaf325

**Published:** 2025-06-06

**Authors:** Yuka Kudo-Nagata, Kazuaki Fukushima, Taro Sugimoto, Tomotake Okuma, Takahiro Hozumi, Toru Motoi, Akifumi Imamura

**Affiliations:** Department of Infectious Diseases, Tokyo Metropolitan Cancer and Infectious Diseases Center Komagome Hospital, Bunkyo-ku, Tokyo, Japan; Department of Infectious Diseases, Tokyo Metropolitan Cancer and Infectious Diseases Center Komagome Hospital, Bunkyo-ku, Tokyo, Japan; Department of Otolaryngology–Head and Neck Surgery, Tokyo Metropolitan Cancer and Infectious Diseases Center Komagome Hospital, Bunkyo-ku, Tokyo, Japan; Department of Musculoskeletal Oncology, Tokyo Metropolitan Cancer and Infectious Diseases Center Komagome Hospital, Bunkyo-ku, Tokyo, Japan; Department of Orthopedic Surgery, Tokyo Metropolitan Cancer and Infectious Diseases Center Komagome Hospital, Bunkyo-ku, Tokyo, Japan; Department of Pathology, Tokyo Metropolitan Cancer and Infectious Diseases Center Komagome Hospital, Bunkyo-ku, Tokyo, Japan; Department of Infectious Diseases, Tokyo Metropolitan Cancer and Infectious Diseases Center Komagome Hospital, Bunkyo-ku, Tokyo, Japan

**Keywords:** EBV, Epstein-Barr virus–associated smooth-muscle tumor, HIV, immunocompromised, opportunistic infection

## Abstract

Epstein-Barr virus–associated smooth-muscle tumor (EBV-SMT) is a rare tumor that occurs in immunosuppressed individuals. Among people living with human immunodeficiency virus (HIV), opportunistic infections are more likely to determine prognosis than EBV-SMT itself. Herein, we describe a patient with HIV whose airway became obstructed due to one of the multiple tumor nodules of EBV-SMT. This 36-year-old woman living with advanced HIV presented with sudden dyspnea. On presentation, she experienced respiratory failure due to airway obstruction, requiring an emergency tracheostomy. Examinations revealed multiple lesions in the right patella, 12th thoracic vertebra, and upper glottis, all diagnosed as EBV-SMT. Despite antiretroviral therapy, surgical interventions were required for disease control. She has remained recurrence free for >3 years after resection. Although EBV-SMT is generally considered an indolent tumor, this case demonstrates that it can cause life-threatening complications, for which a timely multidisciplinary team approach is most crucial.

Epstein-Barr virus (EBV) is associated with several kinds of tumors, including Hodgkin lymphoma, non-Hodgkin lymphoma, and nasopharyngeal carcinoma, in both immunocompetent and immunocompromised individuals [1]. EBV-associated smooth-muscle tumor (EBV-SMT) is classified as a smooth-muscle tumor of uncertain malignant potential [2]. EBV-SMT is a rare disease that primarily affects immunosuppressed individuals. It is classified into 3 major subtypes: the human immunodeficiency virus (HIV)–related type, the posttransplantation type, and the congenital immunodeficiency syndrome–associated type [3, 4].

EBV-SMT can occur anywhere in the body, manifesting preferentially in the central nervous system, visceral organs (eg, gastrointestinal tract, liver, lung, spleen, or adrenal glands), skin, and soft tissues [1, 5–7]. Furthermore, these tumors are often multicentric [8, 9]. Although a structured treatment strategy for EBV-SMT does not yet exist, additional interventions, including a reduction in the dosage of immunosuppressants or surgery, may be performed. Herein, we report a case of life-threatening airway obstruction caused by EBV-SMT with HIV. Our case highlights the necessity of prompt airway management, surgical intervention, and antiretroviral therapy (ART) while emphasizing the importance of a multidisciplinary team approach in the diagnosis and treatment of this disease.

## CASE REPORT

A 36-year-old Thai woman had received a diagnosis of HIV infection following hospitalization for tuberculosis 13 years previously. Her initial CD4 cell count was unknown, and she had discontinued ART on completion of tuberculosis treatment. She had no recent travel history abroad. Six months before her most recent presentation, the patient had experienced right knee pain and visited another hospital. Computed tomography and magnetic resonance (MR) imaging revealed lesions in her right patella with osteolysis. Although no definitive diagnosis was made, she resumed ART (tenofovir alafenamide–emtricitabine and raltegravir) and antituberculosis drug therapy. On her visit to the previous hospital, her CD4 cell count was 107/μL, and her HIV RNA viral load was 1.6 × 10^5^ copies/mL. One month before admission to our hospital, the patient had presented with a hoarse voice, neck pain, and worsening exertional dyspnea. She was referred to our hospital for a comprehensive examination. On the day of her visit, she experienced respiratory failure and loss of consciousness while awaiting examination.

**Figure 1.**
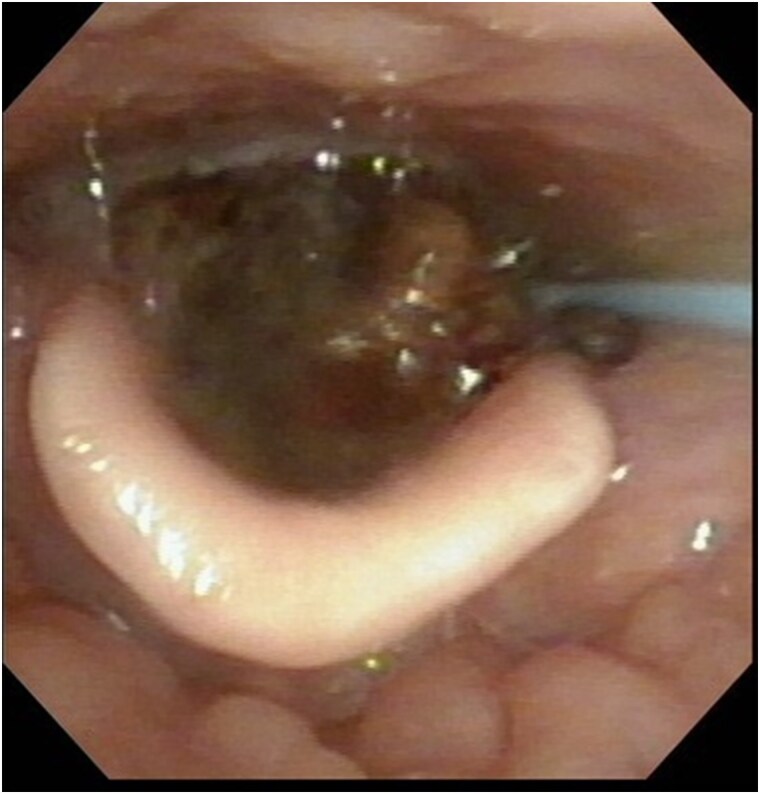
Laryngoscopy demonstrated a hemorrhagic mass approximately 30 mm long above the glottis.

On examination, the patient was diaphoretic and profoundly unwell. Physical examination revealed the following: body temperature, 38.4°C; blood pressure, 120/80 mm Hg; respirations, 28/min; pulse rate, 110/min; and oxygen saturation, 37% with room air and 100% with 10 L/min of oxygen supplementation. Her Glasgow Coma Scale score was E3V2M6. Auscultation revealed bilaterally decreased air entry and the presence of stridor in the patient's throat. A tender, palpable mass was observed above her right knee. Neurological examination showed no sensory or motor impairments in her lower limbs, nor any bladder or bowel dysfunction. Laryngoscopy and emergency tracheostomy were performed immediately to examine the airway obstruction. Laryngoscopy identified a hemorrhagic mass above the glottis (Figure 1), and the glottis itself was obscured by the mass. Blood tests demonstrated no apparent abnormality besides anemia. The patient’s CD4 cell count and HIV RNA viral load were 69/μL and 100 copies/mL, respectively.

**Figure 2.**
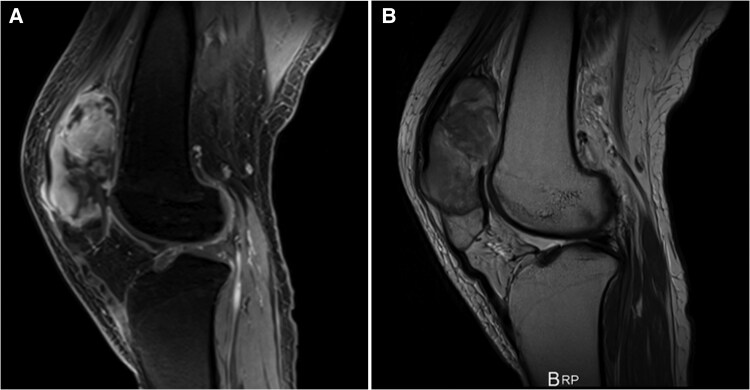
Preoperative contrast-enhanced magnetic resonance (MR) imaging of the right patella revealed a lobulated mass. *A*, Sagittal T1-weighted MR image. *B*, Sagittal T2-weighted MR image.

Computed tomography revealed a nodular lesion within the right forearm flexor muscle group and a 12th thoracic vertebral (T12) spinous process mass causing stenosis of approximately half the dural canal. MR imaging of the spine found an irregularly shaped, intradural, extramedullary mass in the thecal sac at the margin of T12. The mass displayed heterogeneous contrast enhancement and extended posteriorly from the left transverse process to the spinous process. There was severe compression of the spinal cord. Contrast-enhanced MR imaging of the right patella revealed a lobulated mass 6 cm long, centered on the proximal patella with evidence of invasion into the surrounding muscle fascia (Figure 2). The patient later underwent needle biopsy of the right patellar lesion.

Pathological diagnosis of EBV-SMT was based on the histological appearance of spindle-shaped smooth-muscle tumor cell proliferation with lymphocytic infiltration, which was further confirmed by immunohistochemical positivity for α–smooth-muscle actin and Epstein-Barr nuclear antigen 2, as well as positivity for EBV-encoded small RNA (EBER) with in situ hybridization (ISH). At the time these results were obtained, the patient was receiving antituberculosis drugs (isoniazid, ethambutol, streptomycin, levofloxacin, and rifabutin) and ART (dolutegravir and tenofovir alafenamide fumarate–emtricitabine).

**Figure 3.**
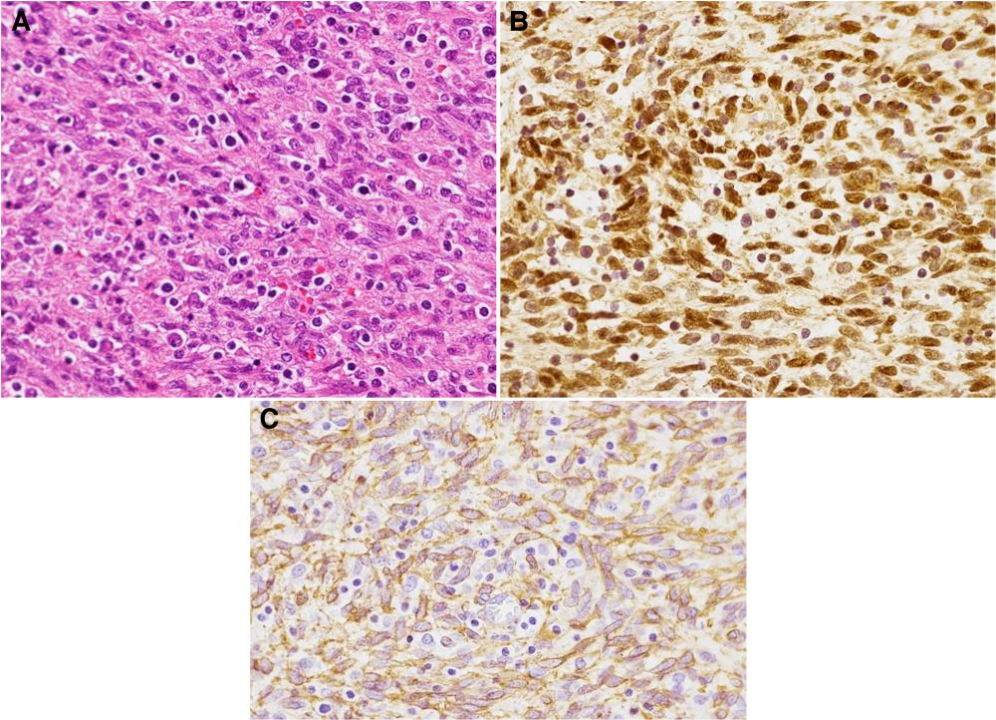
Histological findings for the laryngeal lesion. *A*, Hematoxylin-eosin–stained sections demonstrated interlacing fascicles composed of spindle cells with blunt-ended nuclei. *B*, Tumor cells displayed strong nuclear positivity for Epstein-Barr virus–encoded small RNA (EBER) with in situ hybridization. *C*, Immunohistochemical staining for smooth-muscle actin demonstrated positivity for cytoplasmic granular immunoreactivity in the tumor cells.

The patient underwent decompressive T12 laminectomy with mass excision on hospital day 8. The part of the mass protruding into the spinal canal was 4.0 × 3.7 × 3.0 cm^3^ in size, and the total lesion, including the spinous process, was 7.7 × 6.0 × 4.0 cm^3^. Endoscopic laryngopharyngeal surgery was performed on hospital day 27. Intraoperatively, multiple, soft, elastic lesions were observed. The lesions had a white, membranous surface and measured 22 × 21 × 13 mm^3^, 9 × 8 × 4 mm^3^, and 8 × 4 × 2 mm^3^. ART was continued; however, because the right knee lesion was increasing in size, a tumor resection was performed on hospital day 51. The knee lesion measured 9.5 × 7.2 × 2.5 cm^3^ and consisted of multiple nodules. The 3 specimens were histologically identical and turned out to be EBV-SMT (Figure 3). No abnormal lesions were identified with subsequent esophagogastroduodenoscopy, colonoscopy, or head MR imaging. After the diagnosis was confirmed, antituberculosis drugs were discontinued, and the patient regained the ability to walk independently. No recurrence has been observed during the 3-year follow-up.

Patient written consent was obtained before the publication of this report. All patient-specific information has been anonymized as much as possible. No experiments with human subjects were conducted in this study, so ethics committee approval was not needed.

## DISCUSSION

The combination of patient background, tumoral distribution, and pathological features was a clue to diagnosing EBV-SMT in the present case. Swift airway intervention combined with medium-term management encompassing both ART and appropriate surgical intervention contributed to the patient’s survival. Given her immunosuppressive condition, the multifocality of the lesion, the histological smooth-muscle appearance with inflammatory changes, and the demonstration of EBER by ISH, the pathological diagnosis of EBV-SMT is relatively straightforward [6, 7]. However, the careful exclusion of other inflammatory spindle lesions, such as inflammatory myofibroblastic tumor and leiomyoma/leiomyosarcoma with inflammatory change, may be required for the microscopic observation of hematoxylin-eosin–stained specimens in cases with inadequate clinical information or a single nodular lesion [2, 10]. In such instances, the role of EBER demonstrated by ISH is particularly important.

Surgical resection is the primary therapeutic approach to relieve symptoms associated with the tumors [8]. Other different treatment modalities for EBV-SMT have also been described, including chemotherapy, radiation therapy, and reduction of immunosuppression. However, due to the rarity and unpredictable behavior of these tumors, no consensus on the optimal approach has been established [3, 6]. ART is reinforced in patients with HIV, with the expectation of recovery from immunosuppression, as suggested by previous case series [1, 8]. Some reports indicate that increased CD4 cell counts after ART in combination with surgery have led to disease stabilization or regression [1, 9].

The prognosis is determined by the patient's immune condition [11]. Compared with conventional leiomyosarcoma, EBV-SMT may have a significantly more favorable prognosis [5, 12]. Previous reports of patients with HIV have found that a poor prognosis is more likely to result from a comorbid conditions, such as an opportunistic infection, rather than from the EBV-SMT itself [1, 5, 7, 13]. In contrast, in the present case, a potentially fatal airway obstruction developed due to a delay in diagnosing EBV-SMT. In addition, the progression of the EBV-SMT despite ART posed a serious challenge to treatment, as the patient had mass lesions at multiple anatomic locations, including the spine, bone tissue, and supraglottal area.

Although EBV is not a well-established causative agent of immune reconstitution inflammatory syndrome (IRIS), Chong et al [9] have proposed an IRIS-like phenomenon in HIV-related EBV-SMT, based on criteria suggested by French et al [14]. Caution is warranted in interpreting the present clinical course as IRIS, given that the patient was not observed during the period when IRIS is most likely to manifest (ART having been reinitiated at a different hospital) and due to the limited understanding of the natural history of EBV-SMT, which remains rare. Accordingly, we considered the clinical progression in this case to be attributable to the natural course of EBV-SMT, an IRIS-like reaction, or a combination of both.

Surgical resection was therefore necessary to preserve the patient's life and organ function. The present case demonstrates the importance of considering EBV-SMT in the differential diagnosis for multiple, progressive lesions in patients with poorly controlled HIV. EBV-SMT can cause life-threatening complications, such as airway obstruction or paraparesis, and effective collaboration among specialists is crucial.
